# *In vivo*, *in vitro* and *in silico* correlations of four *de novo SCN1A* missense mutations

**DOI:** 10.1371/journal.pone.0211901

**Published:** 2019-02-08

**Authors:** Andreea Nissenkorn, Yael Almog, Inbar Adler, Mary Safrin, Marina Brusel, Milit Marom, Shayel Bercovich, Daniel Yakubovich, Michal Tzadok, Bruria Ben-Zeev, Moran Rubinstein

**Affiliations:** 1 Service for Rare Disorders, The Edmond and Lily Safra Children’s Hospital, Chaim Sheba Medical Center, Tel HaShomer, Israel; 2 Pediatric Neurology Unit, The Edmond and Lily Safra Children’s Hospital, Chaim Sheba Medical Center, Tel HaShomer, Israel; 3 Sackler School of Medicine, Tel Aviv University, Tel Aviv, Israel; 4 Goldschleger Eye Research Institute, Sackler School of Medicine, Tel Aviv University, Tel Aviv, Israel; 5 Sagol School of Neuroscience, Tel Aviv University, Tel Aviv, Israel; 6 The Arrow Project, The Edmond and Lily Safra Children’s Hospital, Chaim Sheba Medical Center, Tel HaShomer, Israel; 7 Neonatal Intensive Care, Edmond and Lily Safra Children’s Hospital, Chaim Sheba Medical Center, Tel HaShomer, Israel; 8 The Department of Human Molecular Genetics and Biochemistry, Sackler School of Medicine, Tel Aviv University, Tel Aviv, Israel; University of Modena and Reggio Emilia, ITALY

## Abstract

Mutations in the *SCN1A* gene, which encodes for the voltage-gated sodium channel Na_V_1.1, cause Dravet syndrome, a severe developmental and epileptic encephalopathy. Genetic testing of this gene is recommended early in life. However, predicting the outcome of *de novo* missense *SCN1A* mutations is difficult, since milder epileptic syndromes may also be associated. In this study, we correlated clinical severity with functional *in vitro* electrophysiological testing of channel activity and bioinformatics prediction of damaging mutational effects. Three patients, bearing the mutations p.Gly177Ala, p.Ser259Arg and p.Glu1923Arg, showed frequent intractable seizures that had started early in life, with cognitive and behavioral deterioration, consistent with classical Dravet phenotypes. These mutations failed to produce measurable sodium currents in a mammalian expression system, indicating complete loss of channel function. A fourth patient, who harbored the mutation p.Met1267Ile, though presenting with seizures early in life, showed lower seizure burden and higher cognitive function, matching borderland Dravet phenotypes. In correlation with this, functional analysis demonstrated the presence of sodium currents, but with partial loss of function. In contrast, six bioinformatics tools for predicting mutational pathogenicity suggested similar impact for all mutations. Likewise, homology modeling of the secondary and tertiary structures failed to reveal misfolding. In conclusion, functional studies using patch clamp are suggested as a prognostic tool, whereby detectable currents imply milder phenotypes and absence of currents indicate an unfavorable prognosis. Future development of automated patch clamp systems will facilitate the inclusion of such functional testing as part of personalized patient diagnostic schemes.

## Introduction

Dravet syndrome (previously Severe Myoclonic Epilepsy in Infancy, SMEI) is a developmental and epileptic encephalopathy of early childhood with an ominous course [1](www.ilea.org). Children develop normally during the first months but subsequently exhibit unusually severe febrile convulsions before the age of 12 months. Later, various kinds of drug-resistant seizures appear, with or without myoclonus, tending towards the development of *status epilepticus*, especially during febrile illness. Following the onset of epilepsy, developmental delay becomes evident. Cognition continues to deteriorate and ultimately leads to moderate to severe intellectual disability. In parallel with the cognitive decline, communication skills become impaired and autistic features develop, as well as severe behavioral problems [1, 2]. Over 80% of Dravet cases are associated with heterozygous *de novo* mutations in the *SCN1A* gene, which encodes for the alpha subunit of the type I voltage-gated sodium channel (Na_V_1.1), essential for neuronal activity [3].

Prompt and precise diagnosis of Dravet is critical, due to the high risk of *status epilepticus* and sudden unexplained death in epilepsy (SUDEP), and that commonly prescribed antiepileptic sodium channel blockers aggravate Dravet seizures [4]. Hence, in order to address the pressing need for early diagnosis, genetic testing of the *SCN1A* gene is recommended in infants presenting with two or more severe febrile seizures before the age of 12 months [5]. Nevertheless, the interpretation of *SCN1A* genetic analysis is not always trivial.

It has been suggested that the severity of clinical phenotype correlates with the degree of Na_V_1.1 loss-of-function. According to this, severe mutations that result in complete loss of Na_V_1.1 function lead to Dravet syndrome, whereas mutations that only partially reduce Na_V_1.1 activity cause milder phenotypic variants. Included among the latter is GEFS+ (genetic epilepsy with febrile seizure plus), a childhood-onset epilepsy that is well controlled by antiepileptic drugs; and milder forms of Dravet syndrome [6] (www.ilae.org), which manifest lower seizure burden, higher cognitive abilities and lower risk of *status epilepticus* (previously termed SMEI-borderland, SMEB, or intractable childhood epilepsy with generalized tonic-clonic seizures, ICEGTC) [7, 8].

Identification of *de novo SCN1A* nonsense mutations that result in haploinsufficiency allows for immediate Dravet prognosis. However, predicting the damaging effect and clinical significance of *de novo SCN1A* missense mutations is less clear. While roughly half of Dravet patients harbor nonsense mutations, as many as 43% of patients carry *de novo* missense *SCN1A* mutations, leaving prognoses uncertain and families and physicians puzzled [1, 9]. Interestingly, in contrast to *de novo SCN1A* mutations, which are the focus of this report, the clinical outcome of inherited *SCN1A* mutations varies even among kin, probably due to the influence of modifier genes [10–12].

To address the diagnostic challenge of *de novo SCN1A* mutation in patients with early seizure onset, we examined the correlations between clinical presentation, functional ramifications of sodium currents and bioinformatics prediction of expected pathogenicity in four *de novo SCN1A* missense mutations. Our data indicate that current bioinformatics tools cannot predict the severity of epilepsy or cognitive outcome. In contrast, functional studies in a mammalian expression system demonstrate a correlation between the degree of Na_V_1.1 loss-of-function, seizure burden and cognitive outcome. Thus, we propose the use of automated patch clamp systems, which provide rapid and technically easier functional analysis, as part of a personalized prognosis and treatment scheme for patients with *de novo SCN1A* missense mutations.

## Methods

### Patients

Four patients with Dravet syndrome, treated at our pediatric neurology clinic and found to harbor missense mutations in the *SCN1A* gene, were selected for *in vitro* and *in silico* modeling and functional studies. Clinical diagnosis of Dravet syndrome was made based on: a) severe febrile seizures early in life, b) drug-resistant seizures (with or without myoclonus), and c) cognitive deterioration starting after the second year of life [5].

The following clinical data were extracted from patient charts: age, gender, molecular diagnosis, age at onset of seizures, type of seizures, age at onset of febrile seizures, presence of febrile/afebrile *status epilepticus*, antiepileptic drugs used, type of schooling and control of seizures at three time points: two and five years of age, and most-recent visit. Seizure control was defined as the number of seizures per month (excluding myoclonic seizure and atypical absences). Existing EEG records at ages two and five years (±6 months) were used for spectral analysis of the background activity, as described by Holmes et al, 2012 [13]. Two 30 sec recorded segments, devoid of artifacts or epileptiform discharges, during the awake state (with eyes open) were sampled. Recordings were imported to Matlab 6.5 for Windows (Mathworks Inc. Natick, MA, USA) and digitally lowpass filtered with a cut-off frequency of 40 Hz. Power spectrums were generated utilizing the Welch algorithm. Data between 1 and 40 Hz were subjected to calculation of mean frequencies and power band analysis (α/β/δ/θ percentage).

In contrast to standard developmental assessment, in which assorted IQ or DQ scores are evaluated using different methods, we uniformly administered VABS (Vineland Adaptive Behavior Score- Vineland II) performed during regular visits in our neurology clinic. Standard scores for the complete test and for different domains (communication, daily life, socialization and motor skills), as well as V scores for subdomains and maladaptive behavior index were computed and standardized to age according to the Vineland II manual [14].

The study was approved by the institutional review committee at the Sheba Medical Center (IRB- 4870-18-SMC). Subject or parental informed consent was not required since the data was extracted from patient charts while anonymity of the subjects was protected.

### Molecular biology

Human Na_V_1.1 Na^+^ channel α subunit (hNa_V_1.1, NM_006920), inserted into pCDM8 vector, was generously provided by Prof. William A. Catterall (University of Washington, Seattle, WA, USA). The plasmid was propagated in TOP10/P3 cells (Invitrogen, Carlsbad, CA, USA) for > 30 h at 30°C, to minimize spontaneous rearrangement. The entire coding sequence was sequenced after each propagation. Point mutations were introduced using Phusion High-Fidelity DNA Polymerase (New England Biolabs, Ipswich, MA, USA) using the following primers: for Na_V_1.1^G177A^ 5’CTTATAAAAATTATTGCAAGGGCATTCTGTTTAGAAGATTTTACTTTCC and 5’GGAAAGTAAAATCTTCTAAACAGAATGCCCTTGCAATAATTTTTATAAG; for Na_V_1.1^S259R^ 5’CTGTGTTCTGTCTGAGAGTATTTGCTCTAATTGGGCTG and 5’CAGCCCAATTAGAGCAAATACTCTCAGACAGAACACAG; for Na_V_1.1^M1267I^ 5’ CATTTTCATTCTGGAAATACTTCTAAAATGGGTGGCATATGGC and 5’ GCCATATGCCACCCATTTTAGAAGTATTTCCAGAATGAAAATG; and for Na_V_1.1^Q1923R^ 5’ CTGCTGTCATTATTCGGCGTGCTTACAGACGCCACC and 5’ GGTGGCGTCTGTAAGCACGCCGAATAATGACAGCAG.

### Cell culture and transfections

HEK-293 (ATCC CRL-1573) cells were cultured in Dulbecco’s Modified Eagle Medium supplemented with 10% fetal bovine serum, 10 units/mL penicillin and 10μg/mL streptomycin (Biological Industries, Beit-Haemek, Israel), and grown at 37 °C with 5% CO_2_. For electrophysiological recordings, cells were transiently transfected with cDNAs encoding Na_V_1.1^WT^ or mutants (2 μg) and GFP (0.5 μg) in 35mm plates using polyethylenimine (Sigma Aldrich, St. Louis, MO, USA). Recordings were made 2–3 days after transfection. Na_V_1.1^WT^ was transfected with each of the mutants, and each recording session started by recording currents from Na_V_1.1^WT^ in order to verify normal expression.

For Western blot analysis, cells in 60 mm plates were transfected with 6 μg cDNA of Na_V_1.1^WT^ or mutants and cultured for 3 days.

### Electrophysiology

Our electrophysiological recordings were performed as described previously [15] with small changes that are indicated below. Recordings were made using an Axopatch 200B amplifier (Molecular Devices, San Jose, CA, USA), or a Sutter IPA (Sutter Instrument, Novato, CA, USA) amplifier. Data were analyzed using Clampfit (Molecular Devices, San Jose, CA, USA) and IGOR Pro (WaveMetrics, Inc., Lake Oswego, OR, USA). For voltage clamp recording the glass pipettes had resistances of 3–5 MΩ. Capacitive currents were minimized using the amplifier circuitry. We routinely used 75–80% series resistance compensation. The remaining linear capacity and leakage currents were eliminated by P/4 subtraction. The pipette solutions contained (in mM): 140 CsF, 10 NaCl, 1 EGTA, 10 HEPES, 10 glucose, adjusted to pH 7.3 with CsOH. The external solution contained (in mM): 140 NaCl, 20 glucose, 10 HEPES, 1 MgCl_2_, 3 KCl, 1 CaCl_2_, adjusted to pH 7.35 with NaOH. Chemicals were purchased from Sigma-Aldrich (St. Louis, MO, USA) or Fisher Chemical (Waltham, MA USA). The voltage dependence of activation was measured from a holding potential of -120 mV. Cells were depolarized for 20 ms to potentials ranging from -70 to +40 mV in 10 mV increments, and peak inward currents were measured. Conductance (G)-voltage relationships were determined from peak current (I) versus voltage relationships as G = I/(V—V_Rev_), where V was the test potential and V_Rev_ was the extrapolated reversal potential. The voltage dependence of inactivation was measured from a holding potential of -120 mV. Cells were depolarized for 500 ms to potentials ranging from -90 to +50 mV in 10 mV increments, followed by test pulses to -10 mV. Recovery from inactivation was examined by applying a 20 ms conditioning pulse to 0 mV from a holding potential of -120 mV, followed by a recovery interval of variable duration (Δ1 ms) and a test pulse to 0 mV. To examine Na_V_1.1 channel availability, repetitive depolarizations to 0 mV (2 ms long), at 50 Hz, from a holding potential of -70mV, were used.

We attempted a rescue of the function of Na_V_1.1 mutants by applying the specific Na_V_1.1 opener, Hm1a (250 nM) [16], or by pharmacological chaperoning using the Na_V_1.1 modulator N,N'-(1,3-phenylene)bis(2-methylbenzamide), also known as Na_V_1.1-Compound 3a [17]. Na_V_1.1-Compound 3a was shown to increase the firing of fast-spiking interneurons in brain slices and its calculated partition coefficient (clogP of 3.48) is expected to support membrane permeability [17]. Cells were incubated 48–72 h with Na_V_1.1-Compound 3a (30 μM), but the drug was washed out prior to recording to prevent a reduction in peak amplitude.

Hm1a and Na_V_1.1-Compound 3a were purchased from Alomone Labs (Jerusalem, Israel; catalog numbers STH-601 and CMN-002, respectively).

### Western blot

Cells were homogenized in 0.32M sucrose supplemented with protease inhibitors (Sigma-Aldrich, St. Louis, MO, USA), 1mM EDTA and 1mM PMSF, pH 7.4. Crude membrane preparation was produced by centrifugation at 17,000 x g for 90 min. The precipitate (pellet) was solubilized in 150 mM NaCl, 2% Triton X-100 supplemented with protease inhibitors, 1mM EDTA and 1mM PMSF, pH 7.4. A 35 mg aliquot of total protein was separated on Tris-acetate gel (6%) and transferred onto PVDF membrane. After blocking with 5% w/v nonfat dry milk in TBST, the membrane was incubated with anti-Na_V_1.1 antibody (1:200, Alomone Labs, Jerusalem, Israel; catalog number ASC-001) or Alpha 1 Na^+^/K^+^ ATPase (1:200, Alomone Labs, Jerusalem, Israel; catalog number ANP-001), followed by incubation with HRP-conjugated goat anti-rabbit antibody (1:10,000, Sigma-Aldrich, St. Louis, MO, USA). The signal was visualized by chemiluminescent detection using the ECL Detection System.

### Statistical analysis

The data are expressed as mean ± standard error. Normality was tested using the Shapiro-Wilk’s W test. Equal variance was confirmed using the Levene’s test followed by two-tailed Student’s t-test or repeated measures ANOVA, as appropriate. SigmaPlot 12.5 (Systat Software, London, UK) was utilized to calculate statistical measures.

### Bioinformatics assessment of the significance of *SCN1A* missense mutations

The effects of the nonsynonymous amino acid change in the four mutations was predicted using 5 bioinformatics tools: SIFT (Sorting Intolerant From Tolerant algorithm) [18] (http://sift.jcvi.org), MutationAssessor [19] (http://mutationassessor.org), PolyPhen-2 (Polymorphism Phenotyping v2) [20] (http://genetics.bwh.harvard.edu/pph2), Condel (CONsensus DELeteriousness score) [21] (http://bbglab.irbbarcelona.org/fannsdb), and PROVEAN (Protein Variation Effect Analyzer) [22] (http://provean.jcvi.org). In addition, we assessed the pathogenicity of seven control mutations, previously described as causing either Dravet, GEFS+ or febrile seizures [23, 24].

Mutations with a score over the threshold for each bioinformatics tool were considered pathogenic. For MutationAssessor [19], a higher score predicts a more severe mutation (http://mutationassessor.org). Regarding the other assessment tools, a higher score means a greater probability that the prediction of pathogenicity is accurate.

Homology modeling was performed by submitting mutant and wild-type sequences to the SWISS-MODEL Workspace (https://swissmodel.expasy.org). The following templates from the PDB repository (https://www.rcsb.org/pdb) were used for alignment: 4dck1.a (Na_V_1.5) for C terminal mutation and 5xsy.1.A (Na_V_1.2) for the other mutations. Information regarding Na_V_1.1 protein (UniProtKB—P35498) domains and secondary structures were extrapolated from the UniProt Knowledgebase (http://uniprot.org).

## Results

### Patients

**Fig 1:**
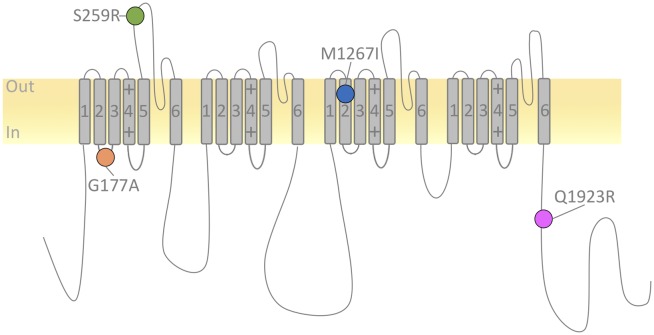
Topology diagram of *SCN1A* missense mutations. Topology diagram of Na_V_1.1 illustrating the location and amino acid substitution of the studied *SCN1A* missense mutations.

Four patients (2 male, 2 female), 5.3–10.8 years of age (mean 8.1), harboring the following *de novo* mutations in the *SCN1A* gene: p.Gly177Ala, p.Ser.259Arg, p.Met1267Ile, and p.Glu1923Arg, were enrolled in this study (Table 1). The locations of these mutations are depicted in Fig 1. The p.Gly177Ala mutation is located within the cytoplasm-facing loop, connecting the S2-S3 segments of the first domain. The p.Ser259Arg and p.Met1267Ile mutations are in the transmembrane domain, within S5 (domain I) and S2 (domain III), respectively. The p.Glu1923Arg mutation is positioned within the intracellular C-terminal domain (Fig 1).

**Table 1:** Clinical characteristics of patients.

Mutation	p.Gly177Ala	p.Ser259Arg	p.Met1267Ile	p.Glu1923Arg
**Age (years)**	6.9	5.3	10.8	9.5
**Gender**	male	female	female	male
**Age at onset of febrile seizures** **(month)**	6	5	4	7
**Age at onset of afebrile seizures** **(month)**	12	12	12	5
**Myoclonic seizures**	no	yes	no	yes
**Status epilepticus**	yes*	yes	yes	yes
**Seizure frequency, age 2 years** **(seizures/month)**	1	0.5	0.5	10
**Seizure frequency, age 5 years** **(seizures/month)**	2	2	1	5
**Seizure frequency, last visit** **(seizures/month)**	1	2	0.25	8
**Antiepileptic treatment** **(last visit)**	VPA, CLB, CBD	VPA, CLB, CBD, VNS	VPA, CLN, STR	VPA, CLB, CBD

*Patient developed severe hypoxic ischemic encephalopathy with left parieto-occipital stroke after prolonged *status epilepticus*

VPA- valproic acid, CLB-clobazam, CLN- clonazepam, STR-stiripentol, CBD- cannabidiol enriched medical marijuana, VNS- vagal nerve stimulator

All patients had severe early febrile seizures, with recurrent febrile and non-febrile *status epilepticus*; one patient (p.Gly177Ala) developed a hypoxic ischemic event with cerebellar stroke and prolonged encephalopathy during *status epilepticus*. Seizure burden differed at 2 years of age, and in particular at 5 years of age (Table 1). A polytherapy regime which included valproic acid and benzodiazepines was used to treat all patients, while three of the patients were also treated with cannabidiol-enriched (CBD 30%) medical marijuana, according to CBD 10 mg/kg/day.

Three patients (p.Gly177Ala, p.Ser259Arg and p.Met1267Ile) had electroencephalography (EEG) recordings at around two and five years of age, suitable for background analysis during the awake state. The mean background frequencies and power band measured in left frontal F3 electrodes are presented in S1 Table. Though this sample size is insufficient for adequate statistical analysis, p.Met1276Ile shows less slowing of background at 2 as well as at 5 years of age, as depicted by higher mean frequency and higher beta power (S1 Table), compared to the other mutations.

All patients were evaluated with Vineland Adaptive Behavior Scale (VABS), which enables the comparison of patient performance at different ages (Table 2). While the patient carrying the p.Met1267Ile mutation scored within normal borderline range, the other patients performed within the low range (Table 2). Interestingly, patients carrying the p.Met1267Ile or p.Ser259Arg mutations performed highest in the communication domain, especially in the receptive language subdomain, in which they were within normal range (V = 13, 13–17 = adequate). Nevertheless, they performed discordantly low in daily living skills (Table 2). Finally, the patient carrying the p.Met1267Ile mutation attends a regular mainstream school with the help of a personal assistant, while the three other children are enrolled in special schooling programs.

**Table 2:** Vineland Adaptive Behavior Score (VABS) in patients.

Mutation		p.Gly177Ala	p.Ser259Arg	p.Met1267Ile	p.Glu1923Arg
**Age at test (years)**		6.9	5.3	10.8	8.5
VABS Standard score* (z score)		56 (0.2%)	65 (1%)	71 (3%)	56 (0.2%)
**Subdomain** Standard Score* (z score)	**Communication**	57 (0.2%)	78 (7%)	79 (8%)	56 (0.2%)
	**Daily living skills**	53 (0.1%)	53 (0.1%)	64 (1%)	57 (0.2%)
	**Socialization**	65 (1%)	70 (2%)	76 (5%)	53 (0.1%)
	**Motor skills**	61 (1%)	67 (1%)	76 (5%)	54 (0.1%)
Maladaptive behavior index**		21	17	20	22

*Adaptive levels according to Standard Score: Low 20–70; Moderately low 71–85; Adequate 86–114; Moderately high 115–129; High 130–160.

** Maladaptive behavior index: Clinically significant 21–24; Elevated 18–20; Average 1–17.

### Biophysical characterization of missense *SCN1A* mutations

**Fig 2:**
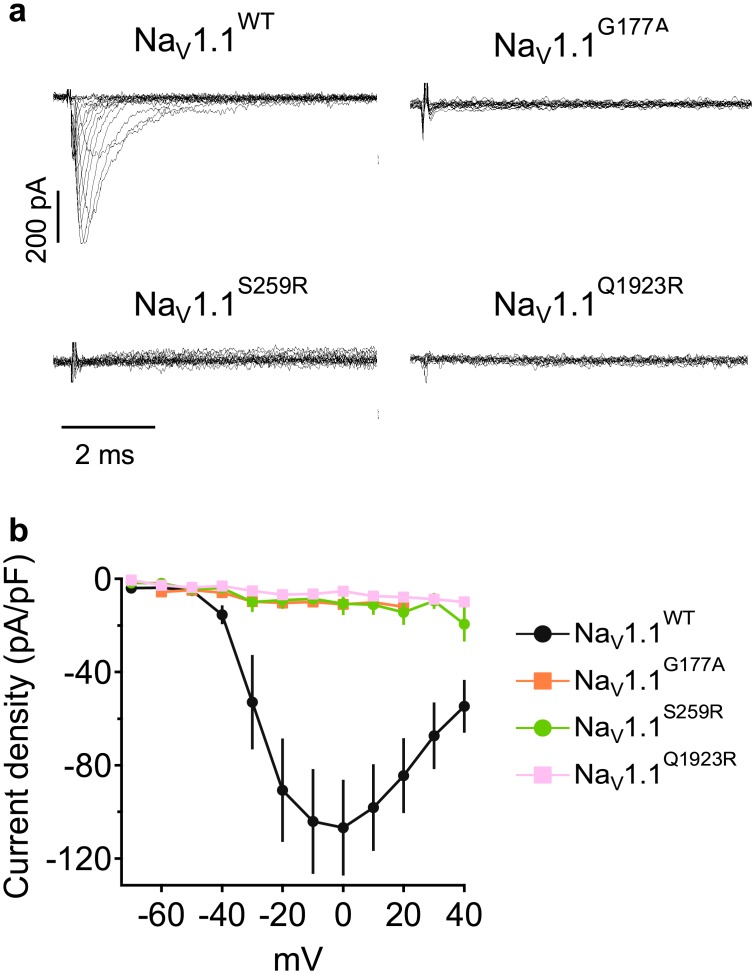
Complete loss of function in missense *SCN1A* mutations. (A) Representative set of sodium current traces from HEK-293 cells expressing Na_V_1.1^WT^, Na_V_1.1^G177A^, Na_V_1.1^S259R^ or Na_V_1.1^Q1923R^. (B) Mean current-voltage (I-V) relationships of sodium current densities. Na_V_1.1^WT^ n = 31; Na_V_1.1^G177A^ n = 10; Na_V_1.1^S259R^ n = 7; Na_V_1.1^Q1923R^ n = 7.

In order to characterize the biophysical ramifications of missense *SCN1A* mutations, we transiently transfected wild-type (WT) and mutant Na_V_1.1 channels into heterologous human HEK-293 cells. The resulting sodium currents were measured using whole-cell voltage clamp. Three of the four missense mutations, p.Gly177Ala, p.Ser259Arg and p.Glu1923Arg, failed to produce detectable sodium currents, suggesting a complete loss of function (Fig 2). We attempted a rescue of function by applying the specific Na_V_1.1 opener, Hm1a [16]. However, while Hm1a increased the amplitude of Na_V_1.1^WT^ channels by 16.8 ± 6.44%, and reduced the inactivation by 28.4 ± 3.57% (S1 Fig), there was no rescue of function for p.Gly177Ala, p.Ser259Arg or p.Glu1923Arg mutations (S1 Fig).

We further examined the membrane expression of Na_V_1.1 channels in crude membrane preparation using western blot (S2 Fig). The expression of the p.Gly177Ala was reduced, potentially indicating defects in protein synthesis, trafficking or stability, while the expression of the other mutants was comparable to that of Na_V_1.1^WT^ (S2 Fig). Our membrane preparation does not differentiate between the plasma membrane and other cellular membranes, including those of the endoplasmic reticulum. Thus, with or without Hm1a, the absence of currents (Fig 2 and S1 Fig) may be a result of defective trafficking, defective gating or both.

We further evaluated the effect of a pharmacological chaperoning by incubating the transfected cells with Na_V_1.1-Compound 3a, which is a modulator of Na_V_1.1 activity [17]. Under these conditions, we were able to record small currents in some cells (S3A Fig), but the rescue was still minimal (S3 Fig).

**Fig 3:**
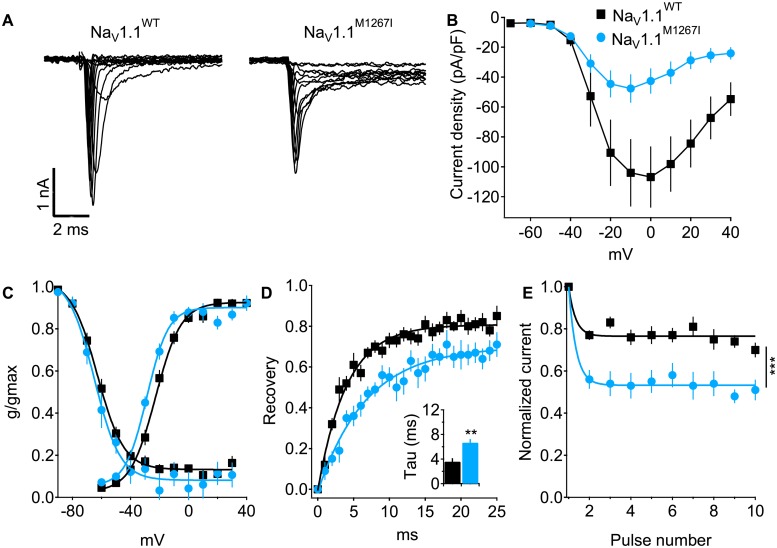
Partial loss of function in the p.Met1269Ile mutations. (A) Representative sodium current traces from HEK-293 expressing Na_V_1.1^WT^ or Na_V_1.1^M1267I^. (B) Mean current-voltage (I–V) relationships of current densities. (C) Voltage dependence of activation (right curves: V_1/2_ of -24.1 ± 1.5 mV for Na_V_1.1^WT^ and -31.4 ± 1.3 mV for Na_V_1.1^M1267I^, p < 0.01; Na_V_1.1^WT^ n = 31; Na_V_1.1^M1267I^ n = 16) or the voltage dependence for steady-state fast inactivation (left curves, V_1/2_ of -63.6 ± 2 mV for Na_V_1.1^WT^ and -65.2 ± 2.5 mV, for Na_V_1.1^M1267I^ p > 0.05; Na_V_1.1^WT^ n = 26; Na_V_1.1^M1267I^ n = 15). (D) Recovery from fast inactivation (Na_V_1.1^WT^ n = 8; Na_V_1.1^M1267I^ n = 7). (E) Normalized currents during 10 depolarizations from -70 mV to 0 mV at 50 Hz (Na_V_1.1^WT^ n = 9; Na_V_1.1^M1267I^ n = 9).

In contrast, expression of the p.Met1267Ile mutation resulted in sodium currents that were similar in amplitude to Na_V_1.1^WT^ without normalization of cell capacitance (S4A Fig), but reduced after this normalization (Fig 3A and 3B). Persistent sodium currents, measured at the end of the 20 ms depolarization, were not statistically different between Na_V_1.1^WT^ and Na_V_1.1^M1267I^ (S4B Fig). There was a shift toward hyperpolarization in the voltage dependency of activation (Fig 3C), and a marked slowing of the recovery from fast inactivation (Fig 3D). Moreover, while the amplitude of Na_V_1.1^WT^ channels declined by ~20% during repetitive depolarizations at 50 Hz, the reduction in channel availability was doubled in the Na_V_1.1^M1267I^ (Fig 3E). Together, our functional data indicate that the p.Met1267Ile mutation confers partial loss of function effect. Nevertheless, similarly to the effect on Na_V_1.1^WT^, application of the Hm1a toxin increased the peak currents of Na_V_1.1^M1267I^ by 21.13 ± 8% and reduced its inactivation by 21.2 ± 7% (S1 Fig).

Together, our functional analyses revealed complete loss of function in the p.Gly177Ala, p.Ser259Arg and p.Glu1923Arg mutations, while the p.Met1267Ile mutation demonstrated a partial loss of function.

### Bioinformatics assessment of pathogenicity of *SCN1A* mutations

The predicted outcome of these mutations was further analyzed using five bioinformatics tools (Table 3). While all four mutations were predicted to be damaging, the predicted scores with SIFT, PolyPhen-2 and Condel were similar for all four mutations. PROVEAN scores were inconsistent (Table 3). In contrast, MutationAssessor predicted a lower impact on protein function for the p.Met1267Ile mutation compared to the three other mutations (Table 3). To further examine the ability of MutationAssessor to correctly predict the outcome of *SCN1A* mutations, we tested additional missense mutations [23, 24] (Table 3). This resulted in a lack of correlation between the predicted dampening effect and the clinical severity (Table 3).

**Table 3:** Prediction of mutation impact according to bioinformatics tools in patients and controls.

	G177A Dravet	S259R Dravet	M1267I Dravet	Q1923R Dravet	R859C GEFS+Dravet	W1204R GEFS+ Dravet	V1336I GEFS+ Dravet	M1664K GEFS+ Dravet	Y790C GEFS+	M145T FS	M956T FS
**SIFT**	**0**	**0**	**0**	**0**	0	0	0	0	0	0	0
**PolyPhen-2**	**0.99**	**0.99**	**0.99**	**0.99**	0.99	0.99	0.99	0.99	0.97	0.91	0.99
**Mutation Assessor**	**3.975**	**3.505**	**2.4**	**3.56**	4.44	3.76	3.30	3.12	3.58	3.53	3.41
**Condel**	**0.72**	**0.68**	**0.6**	**0.68**	0.75	0.71	0.67	0.66	0.69	0.68	0.67
**PROVEAN**	**-5.77**	**-4.88**	**-2.73**	**-3.68**	-7.34	-13.37	-0.95**	-5.22	-8.09	-5.48	-5.80
**Significance**	**Damaging**	**Damaging**	**Damaging**	**Damaging**	Damaging	Damaging	Damaging**	Damaging	Damaging	Damaging	Damaging

In bold, *SCN1A* mutations that were studied here, GEFS+ genetic epilepsy with febrile seizures plus, FS-febrile seizures.

SIFT [18], threshold for pathogenic mutation ≤ 0.05 damaging; PolyPhen-2 [20], > 0.85 probably damaging; Mutation Assessor [19], 0.8–1.9 low impact, 1.9–3.5 medium impact, > 3.5 high impact; Condel [21], >0.469 deleterious; PROVEAN [22], < -2.5 deleterious

** V1336I mutations neutral/tolerated in PROVEAN but damaging in other tools

Moreover, homology modeling failed to reveal any derangement in secondary or tertiary structure in all mutants vs. wild-type protein (S5 Fig). Thus, current bioinformatics servers are unable to predict the functional or clinical outcome of *SCN1A* missense mutations.

## Discussion

The incidence of Dravet Syndrome is 1 in 16,000 births [25], with most cases attributable to *de novo SCN1A* mutations. With today’s routine use of genetic analysis, *SCN1A* mutations are usually detected by the end of the first year of life [5]. Nevertheless, the clinical outcome of *SCN1A* missense mutations is difficult to predict, as these *de novo* mutations can also lead to milder forms of epilepsy [26]. In this study, we correlated the clinical presentation, biophysical significance and bioinformatics prediction of four *de novo SCN1A* missense mutations. Notably, while multiple modes of bioinformatics analysis failed to predict the severity of the mutation, sodium current recordings, acquired using patch clamp analysis in a mammalian expression system, demonstrated a correlation between the degree of Na_V_1.1 loss of function, seizure burden and adaptive abilities.

### Prompt and correct diagnosis of Dravet is critical

Correct and prompt diagnosis is critical, allowing the establishment of early treatment with appropriate anti-seizure medication, as well as aggressive rescue plans in episodes of *status epilepticus* [5]. To facilitate this, sequencing of the *SCN1A* gene is recommended in children with a clinical picture suggestive of Dravet syndrome [5, 27]. While the degree of Na_V_1.1 loss of function is considered correlative with the severity of clinical phenotype [6, 26], prognosis in *de novo* missense *SCN1A* mutations generally awaits the presentation of developmental delay due to the challenges remaining in correctly foretelling the functional outcome of these mutations.

Recent literature reports that the age of onset of seizures is a reliable prognostic factor that can differentiate between Dravet and GEFS+ [28]. However, this predictor cannot be used to differentiate between severe and milder Dravet cases. Our patients similarly presented febrile seizure before the age of 12 months. Yet, the patient with the p.Met1267Ile mutation had milder disease outcome, demonstrated by better pharmacological control of seizures, faster EEG background and adaptive function within normal borderline range. Thus, additional predictors are needed.

### Current bioinformatics tools fail to predict clinical severity

Bioinformatics tools and servers are widely used to predict the significance of mutations in various genes. In this study, we compared six available bioinformatics tools that are routinely used in predicting disease outcome (Table 3 and S5 Fig). While all of the mutations were suggested to be pathogenic, none of these tools differentiated between the milder p.Met1267Ile mutation and the other, more severe mutations. Furthermore, these servers were unable to distinguish between Dravet mutations, febrile seizures and GEFS+ mutations (Table 3).

These servers, including SIFT [18], PolyPhen-2 [20], Condel [21] and PROVEAN [22], calculate the probability that a missense mutation will be pathogenic, rather than specify the impact on protein function. It is therefore not surprising that predicting the clinical outcome lies outside of their reach. In contrast, MutationAssessor [19] analyzes the functional effect of a mutation, and thus was assessed for its ability to predict the functional severity of the mutations. This tool was originally designed to predict the impact of cancer-inducing genes rather than membrane-expressed ion channels, and it too failed to differentiate between mild and severe *SCN1A* mutations (Table 3).

Moreover, we tried to use the readily available tools for predicting secondary and tertiary protein structure to compare wild-type versus mutant Na_V_1.1 channels, but failed to reveal any misfolding (S5 Fig). These mathematical models predict protein structure by computing the position of atoms, which confers minimal energetic level and maximal stability to the protein structure, and aligning them against a template of homologous proteins with known crystallographic or MRI structure [29]. While these automated pipelines, such as SWISS-MODEL are user-friendly and can predict different functional domains, they are probably not sensitive enough to perceive perturbations caused by single point mutations. Thus, despite the high accessibility of bioinformatics servers to clinicians, they fail to predict the future clinical course.

### Functional studies of *SCN1A* mutations in expression systems can predict the degree of pathogenicity

In this study, three of the mutations tested (p.Gly177Ala, p.Ser259Arg and p.Glu1923Arg) indicated complete loss-of-function effect, while the p.Met1267Ile mutation demonstrated complex biophysical changes, likely to cause partial loss of function. The degree of *in vitro* loss of function correlates well with the severity of clinical phenotypes. Thus, we propose that lack of functional channels can predict an unfavorable outcome of *de novo SCN1A* missense mutations, while detectable currents, regardless of their biophysical properties, indicate milder phenotypes. Corroborating this, previous electrophysiological studies indexed in the *SCN1A* mutation database (http://www.gzneurosci.com/scn1adatabase) reported complete loss-of-function in 11 out of 19 Dravet missense mutations listed [30–36]. While detailed clinical information is missing for most of these mutations, thereby preventing the differentiation between classical and milder forms of Dravet [30, 32, 35], Volkers et al (2011) describe lower seizure burden in a functional mutation (p.Arg865Gly) versus a nonfunctional channel (p.Arg946Cys) [31]. Furthermore, febrile seizures and GEFS+ associated *SCN1A* missense mutations produce detectable current with an amplitude of at least 100 pA [23].

We thus propose the use of functional tests, using patch clamp analysis, as part of the diagnosis process. To date, patch clamp experiments are not readily available for clinicians. However, automated patch clamp set-ups are widely used in drug discovery companies, offering rapid and simple functional analysis of ion channel activity [37]. Therefore, functional analysis of *de novo* missense *SCN1A* mutations, using automated patch clamp systems, can be implemented as part of the personalized diagnostic procedure and may soon become as routine and straightforward as genetic analysis.

Our attempts to rescue the function of Na_V_1.1 mutants G177A, S259R and Q1923R by pharmacological chaperoning (Na_V_1.1-Compound 3a [17]) or by application of Hm1a were unsuccessful (S1 and S3 Figs). In contrast, the Hm1a toxin was able the increase the currents and reduce the inactivation of the M1267I mutation (S1 Fig). With further development of Na_V_1.1 openers for clinical use [38], functional analysis may provide information about the appropriate dosages. Mutations causing partial loss of function might foreseeably require lower dosages than mutations that confer complete loss of function.

To gain mechanistic insights about pathogenicity, neuronal systems such as cultured neurons or induced pluripotent stem cell (iPSC)-derived neurons may be needed. Moreover, genetic background greatly affects disease severity, especially in the case of inherited missense *SCN1A* mutations [10]. Indeed, three of the eight functional Dravet associated mutations (~40%) are familial mutations (p.Arg1648Cys [39], p.Arg1657Cys [33] p.Met1852Thr [36]), with mild and severe phenotypes within the same kindred. To study the role of modifier genes, mouse models and patient-derived neurons may be useful [40–47]. Foreseeably at this time, these preparations are neither automated nor likely to become accessible in the near future as part of routine clinical diagnosis.

### Clinical assessment of Dravet patients

Assessment of disease severity is challenging, as seizure burden and cognitive abilities of Dravet patients change with age. We compared the frequency of seizures and EEG background activity of patients at two and five years of age. Additionally, we administered the VABS test which, unlike other IQ or DQ tests, allows comparison of adaptive daily functions at different ages, irrespective of developmental level and cooperation. VABS has previously been used in two studies assessing cognition and behavior in children with Dravet [48, 49]. This type of assessment suggested a distinctive profile for these children, with higher scores on the socialization domain, as compared to communication or living skills. While our cohort is small, our patients scored higher in the communication domain. Interestingly, two patients (p.Ser259Arg and p.Met1267Ile) scored within the normal range in the verbal receptive subdomain and discordantly low in daily living (personal, domestic and community) domain (Table 2). These results reinforce the notion that Dravet patients may have a uniquely identifying functional profile, which distinguishes them from children with autism or intellectual disability. Our findings warrant further evaluation of larger patient cohorts using VABS.

## Conclusion

Our data indicate that, while currently available bioinformatics tools are insufficient for predicting the severity of epilepsy and cognitive outcome, functional studies in mammalian expression systems may foretell the severity of *de novo SCN1A* missense mutations. Future technological development of automated patch-clamp set-ups, suitable for clinical use, may facilitate the inclusion of electrophysiological tests as part of a personalized diagnosis and treatment scheme for patients carrying *de novo SCN1A* mutations.

## Supporting information

S1 Table Quantitative EEG evaluation of at two and five years of age. Mean background frequencies and power band in left frontal F3 montage are depicted. No EEG`s are available for the patient with the p. Glu1923Arg mutation.
(PDF)

S1 Fig Hm1a does not rescue the activity of Na_V_1.1 mutants G177A, S259R and Q1923R. Currents from HEK-293 cells transiently expressing Na_V_1.1 channels in the absence (black) or presence (red) of 250 nM Hm1a. Currents were elicited by depolarizations to 0 mV from a holding potential of -120 mV. We calculated the effect of Hm1a as the difference in peak amplitude and inactivation (current at the end of the pulse / peak current) before and after Hm1a application. Hm1a increased the peak amplitude of Na_V_1.1^WT^ by 16.8 ± 6.44%, and reduced the inactivation by 28.4 ± 3.57%. Comparable effects were measured for Na_V_1.1^M1267I^ with 21.13 ± 8% increase of peak amplitude and 21.2 ± 7% reduction in inactivation.
(PDF)

S2 Fig Na_V_1.1 expression in total membranes. Representative immunoblots of Na_V_1.1 expression in total membranes. The rightmost lane represents untransfected HEK-293 cell (-). The lower panel is an Na^+^/K^+^ ATPase loading control. The bar graph is a quantification of the normalized expression of three independent experiments. For each lane, Na_V_1.1 protein expression was first corrected to the relative expression of the Na^+^/K^+^ ATPase loading control. Next, in order to combine different experiments, the data were further normalized to the corrected expression of Na_V_1.1^WT^ in each experiment.
(PDF)

S3 Fig Pharmacological chaperoning. The effect of pharmacological chaperoning. The cells were incubated for 48-72h with 30 μM of the Na_V_1.1 modulator, N,N'-(1,3-phenylene)bis(2-methylbenzamide), also known as Na_V_1.1-Compound 3a. The drug was not included in the external recording solution to prevent a reduction in peak amplitude. (A) Representative set of sodium current traces from HEK-293 cells expressing Na_V_1.1^WT^, Na_V_1.1^G177A^, Na_V_1.1^S259R^ or Na_V_1.1^Q1923R^. (B) Mean current-voltage (I-V) relationships of sodium current densities. Na_V_1.1^WT^ n = 12; Na_V_1.1^G177A^ n = 10; Na_V_1.1^S259R^ n = 7; Na_V_1.1^Q1923R^ n = 7. (C) Average current densities at -10 mV, with or without (Cnt) incubation with Na_V_1.1-Compound 3a.
(PDF)

S4 Fig Na_V_1.1^WT^ and Na_V_1.1^M1267I^. (A) Mean current–voltage (I–V) relationships of peak currents for Na_V_1.1^WT^ and Na_V_1.1^M1267I^, not normalized to cell capacitance. (B) Persistent currents (% of peak currents) measured at the end of 20 ms depolarization to 0 mV.
(PDF)

S5 Fig Homology modeling. Homology modeling of WT (A,C,E,G) and mutant Na_V_1.1 (B,D,F,H).
(PDF)
