# Supplementary material for: In vivo, in vitro and in silico correlations of four de novo SCN1A missense mutations
Source: PLoS One. 2019 Feb 8;14(2):e0211901. doi: 10.1371/journal.pone.0211901 (PMC6368302; doi:10.1371/journal.pone.0211901)
Supplement: S1 Table — Mean background frequencies and power band in left frontal F3 montage are depicted. No EEG`s are available for the patient with the p. Glu1923Arg mutation. (PDF) [file pone.0211901.s001.pdf]

S1 Table. Quantitative EEG evaluation at two and five years of age.

**Mutation**	**p.Gly177Ala**		**p.Ser259Arg**		**p.Met1267Ile**	
**Age (years)**	2	5	2	5	2	5
**Mean frequency**	9.46	9.18	6.02	10.08	14.06	13.66
**Delta power (%)**	19.24	17.48	44.53	22.34	20.15	20.01
**Theta power (%)**	20.24	25.24	25.2	29.34	13.95	13.73
**Alpha power (%)**	25.49	22	22.86	23.33	22.88	27.45
**Beta power (%)**	35.02	35.28	7.41	24.84	43.02	38.74

Mean background frequencies and power band in left frontal F3 montage are depicted. No EEG`s are available for the patient with the p. Glu1923Arg mutation.
