# Supplementary material for: In vivo, in vitro and in silico correlations of four de novo SCN1A missense mutations
Source: PLoS One. 2019 Feb 8;14(2):e0211901. doi: 10.1371/journal.pone.0211901 (PMC6368302; doi:10.1371/journal.pone.0211901)
Supplement: S1 Fig — Currents from HEK-293 cells transiently expressing NaV1.1 channels in the absence (black) or presence (red) of 250 nM Hm1a. Currents were elicited by depolarizations to 0 mV from a holding potential of -120 mV. We calculated the effect of Hm1a as the difference in peak amplitude and inactivation (current at the end of the pulse / peak current) before and after Hm1a application. Hm1a increased the peak amplitude of NaV1.1WT by 16.8 ± 6.44%, and reduced the inactivation by 28.4 ± 3.57%. Comparable effects were measured for NaV1.1M1267I with 21.13 ± 8% increase of peak amplitude and 21.2 ± 7% reduction in inactivation. (PDF) [file pone.0211901.s002.pdf]

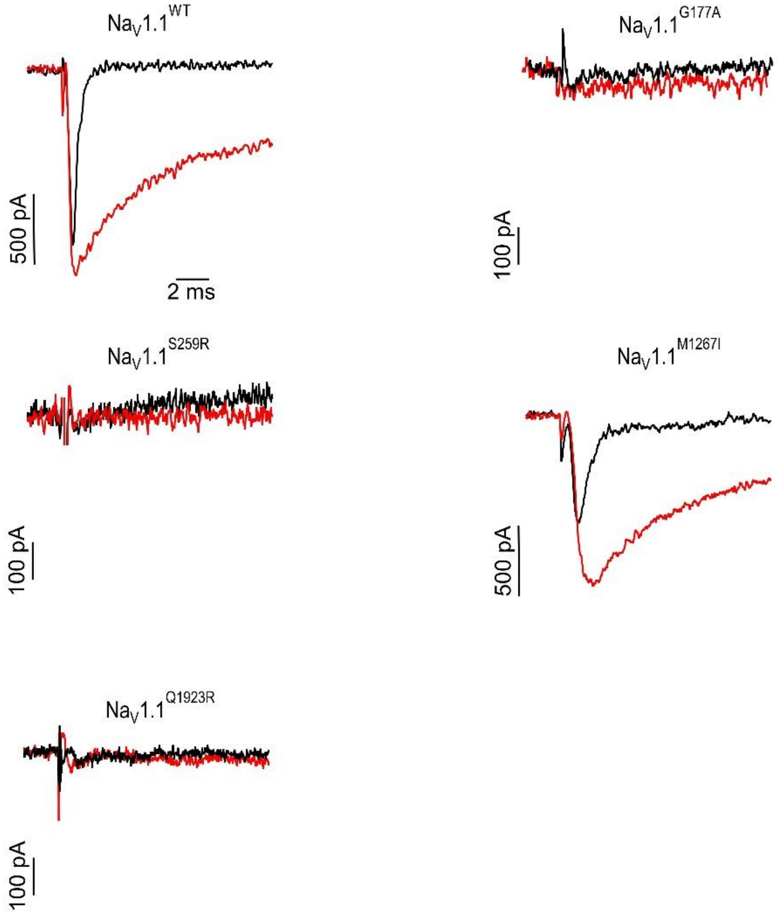

S1 Fig. Hm1a does not rescue the activity of Na$_V$1.1 mutants G177A, S259R and Q1923R. Currents from HEK-293 cells transiently expressing Na$_V$1.1 channels in the absence (black) or presence (red) of 250 nM Hm1a. Currents were elicited by depolarizations to 0 mV from a holding potential of -120 mV. We calculated the effect of Hm1a as the difference in peak amplitude and inactivation (current at the end of the pulse/peak current) before and after Hm1a application. Hm1a increased the peak amplitude of Na$_V$1.1^{WT} by 16.8 ± 6.44 %, and reduced the inactivation by 28.4 ± 3.57 %. Comparable effects were measured for Na$_V$1.1^{M1267I} with 21.13 ± 8 % increase of peak amplitude and 21.2 ± 7 % reduction in inactivation.
