# Supplementary material for: In vivo, in vitro and in silico correlations of four de novo SCN1A missense mutations
Source: PLoS One. 2019 Feb 8;14(2):e0211901. doi: 10.1371/journal.pone.0211901 (PMC6368302; doi:10.1371/journal.pone.0211901)
Supplement: S2 Fig — Representative immunoblots of NaV1.1 expression in total membranes. The rightmost lane represents untransfected HEK-293 cell (-). The lower panel is an Na+/K+ ATPase loading control. The bar graph is a quantification of the normalized expression of three independent experiments. For each lane, NaV1.1 protein expression was first corrected to the relative expression of the Na+/K+ ATPase loading control. Next, in order to combine different experiments, the data were further normalized to the corrected expression of NaV1.1WT in each experiment. (PDF) [file pone.0211901.s003.pdf]

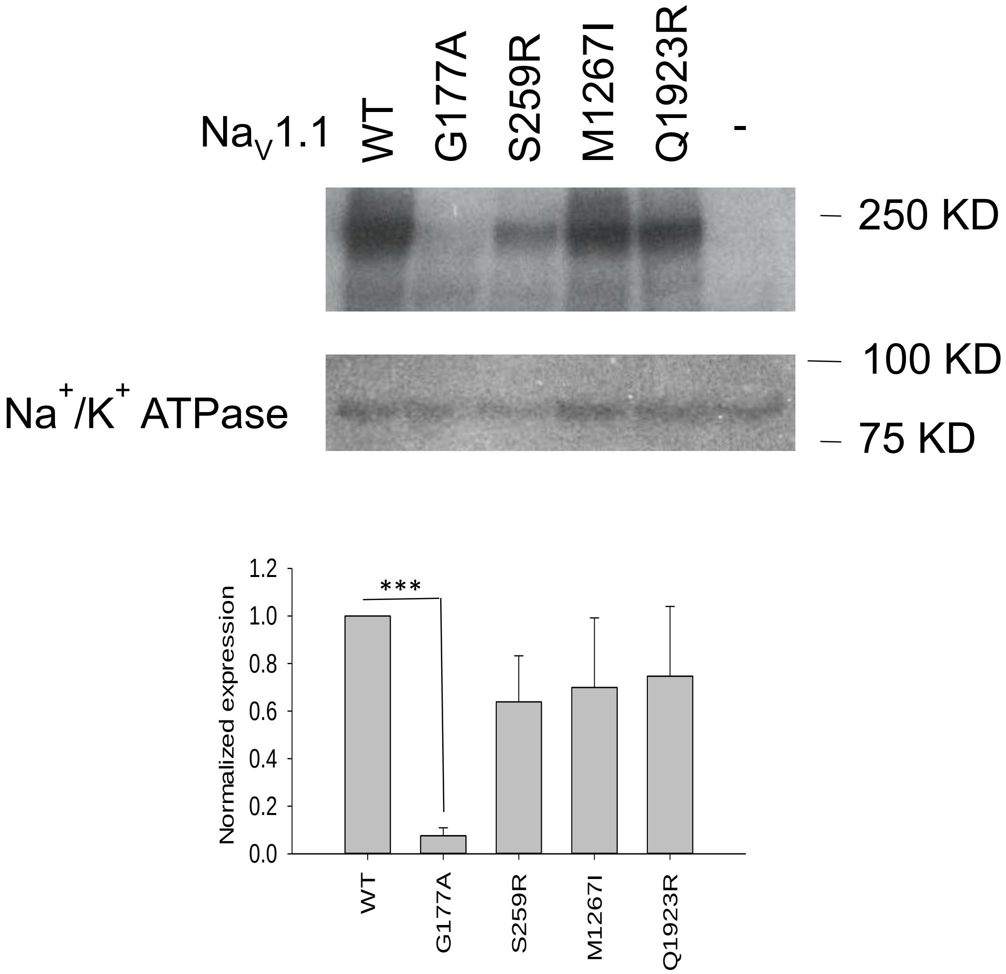

S2 Fig. Na$_V$1.1 expression in total membranes. Representative immunoblots of Na$_V$1.1 expression in total membranes. The rightmost lane represents untransfected HEK-293 cell (-). The lower panel is the Na$^+$/K$^+$ ATPase loading control**.** The bar graph is a quantification of the normalized expression of three independent experiments. For each lane, Na$_V$1.1 protein expression was first corrected to the relative expression of the Na$^+$/K$^+$ ATPase loading control. Next, in order to combine different experiments, the data were further normalized to the corrected expression of Na$_V$1.1^{WT} in each experiment.
