# Supplementary material for: In vivo, in vitro and in silico correlations of four de novo SCN1A missense mutations
Source: PLoS One. 2019 Feb 8;14(2):e0211901. doi: 10.1371/journal.pone.0211901 (PMC6368302; doi:10.1371/journal.pone.0211901)
Supplement: S3 Fig — The effect of pharmacological chaperoning. The cells were incubated for 48-72h with 30 μM of the NaV1.1 modulator, N,N'-(1,3-phenylene)bis(2-methylbenzamide), also known as NaV1.1-Compound 3a. The drug was not included in the external recording solution to prevent a reduction in peak amplitude. (A) Representative set of sodium current traces from HEK-293 cells expressing NaV1.1WT, NaV1.1G177A, NaV1.1S259R or NaV1.1Q1923R. (B) Mean current-voltage (I-V) relationships of sodium current densities. NaV1.1WT n = 12; NaV1.1G177A n = 10; NaV1.1S259R n = 7; NaV1.1Q1923R n = 7. (C) Average current densities at -10 mV, with or without (Cnt) incubation with NaV1.1-Compound 3a. (PDF) [file pone.0211901.s004.pdf]

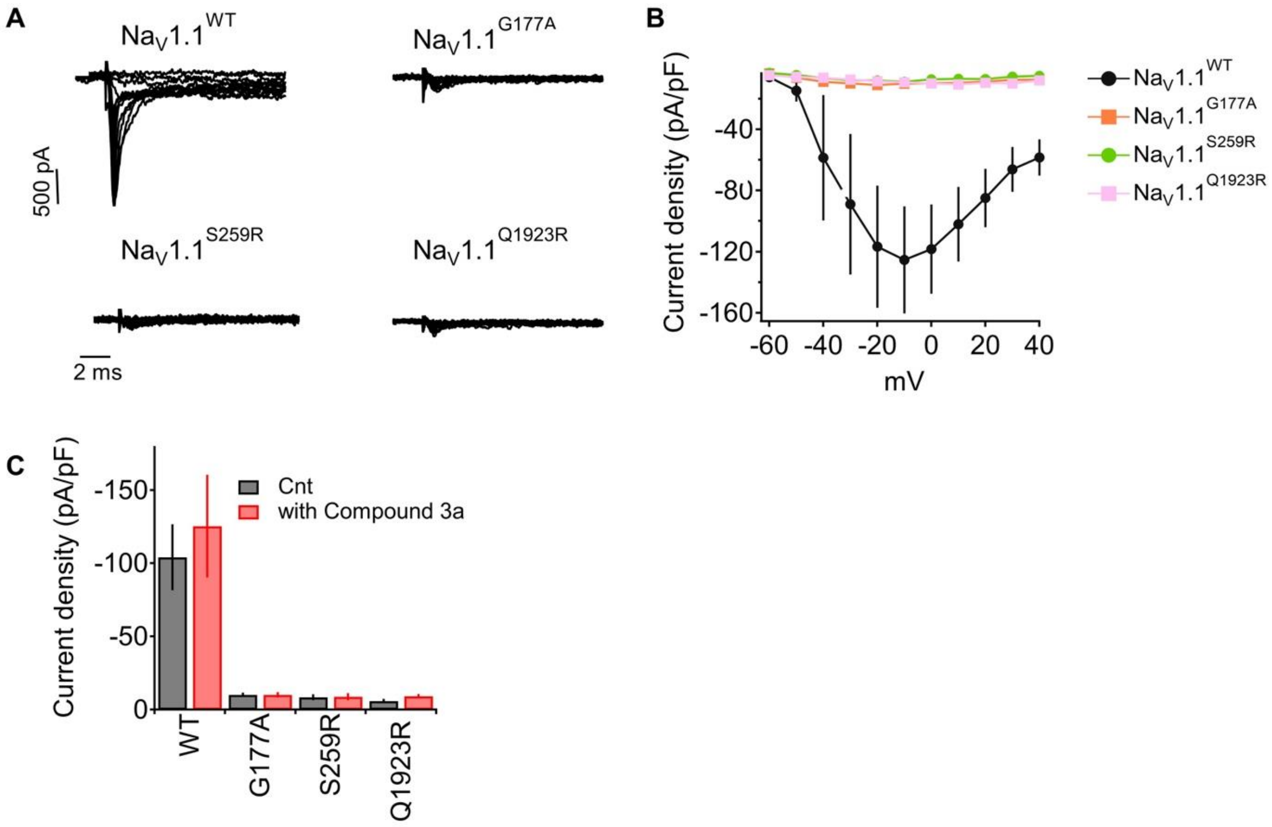

S3 Fig. Pharmacological chaperoning. The cells were incubated for 48-72h with 30 μM of the $Na_V1.1$ modulator, N,N'-(1,3-phenylene)bis(2-methylbenzamide), also known as $Na_V1.1$-Compound 3a. The drug was not included in the external recording solution, to prevent a reduction in peak amplitude. (A) Representative set of sodium current traces from HEK-293 cells expressing $Na_V1.1^{WT}$, $Na_V1.1^{G177A}$, $Na_V1.1^{S259R}$ or $Na_V1.1^{Q1923R}$. (B). Mean current-voltage (I-V) relationships of sodium current densities. $Na_V1.1^{WT}$ n=12; $Na_V1.1^{G177A}$ n=10; $Na_V1.1^{S259R}$ n=7; $Na_V1.1^{Q1923R}$ n=7. (C) Average current densities at -10 mV, with or without (Cnt) incubation with $Na_V1.1$-Compound 3a.
