# Supplementary material for: In vivo, in vitro and in silico correlations of four de novo SCN1A missense mutations
Source: PLoS One. 2019 Feb 8;14(2):e0211901. doi: 10.1371/journal.pone.0211901 (PMC6368302; doi:10.1371/journal.pone.0211901)
Supplement: S4 Fig — (A) Mean current–voltage (I–V) relationships of peak currents for NaV1.1WT and NaV1.1M1267I, not normalized to cell capacitance. (B) Persistent currents (% of peak currents) measured at the end of 20 ms depolarization to 0 mV. (PDF) [file pone.0211901.s005.pdf]

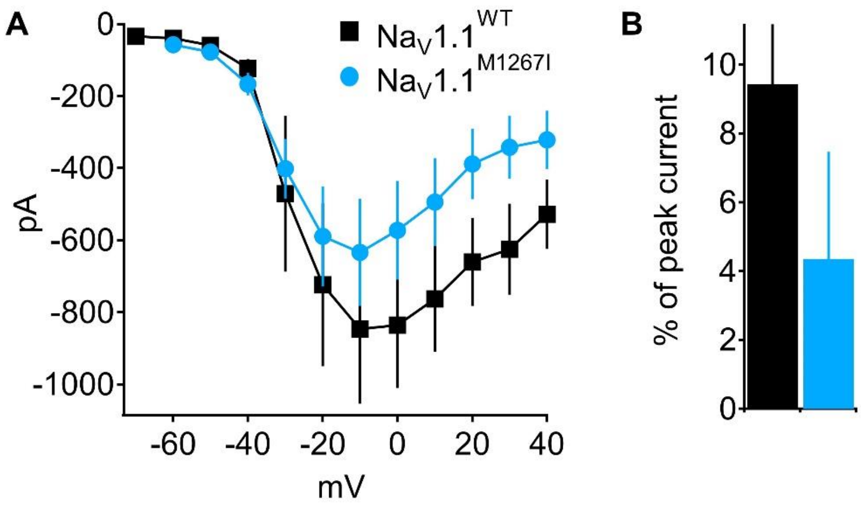

S4 Fig. Na$_V$1.1^{WT} and Na$_V$1.1^{M1267I}. (A) Mean current-voltage (I–V) relationships of peak currents for Na$_V$1.1^{WT} and Na$_V$1.1^{M1267I}, not normalized to cell capacitance. (B) Persistent currents (% of peak currents) measured at the end of 20 ms depolarization to 0 mV.
