# Supplementary figures and images for: In vivo, in vitro and in silico correlations of four de novo SCN1A missense mutations
Source: PLoS One. 2019 Feb 8;14(2):e0211901. doi: 10.1371/journal.pone.0211901 (PMC6368302; doi:10.1371/journal.pone.0211901)

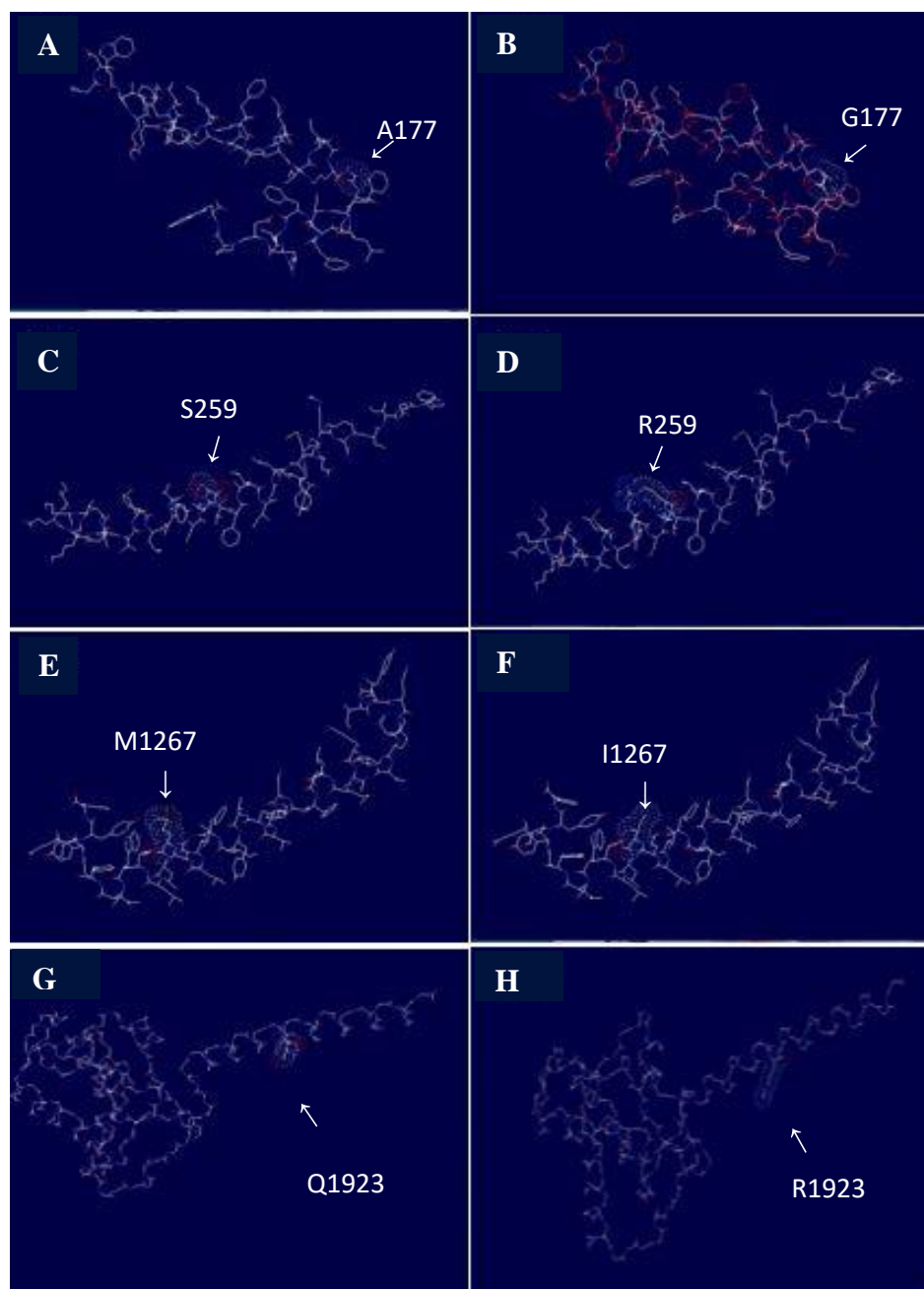

**S5 Fig. Homology modeling.** Homology modeling of WT (A,C,E,G) and mutant Nav1.1 (B,D,F,H).

Supplement: S5 Fig — Homology modeling of WT (A,C,E,G) and mutant NaV1.1 (B,D,F,H). (PDF) [file pone.0211901.s006.pdf]
